# Ten-Year (1999–2009) Epidemiological and Virological Surveillance of Influenza in South Italy (Apulia)

**DOI:** 10.1155/2010/642492

**Published:** 2010-06-22

**Authors:** Annamaria Campa, Manuela Quattrocchi, Marcello Guido, Giovanni Gabutti, Cinzia Germinario, Antonella De Donno, The Influenza Collaborative Group

**Affiliations:** ^1^Laboratory of Hygiene, Department of Biological and Environmental Sciences and Technologies (DiSTeBA), University of Salento, Via Prov. Le Lecce-Monteroni 73100 Lecce, Italy; ^2^Department of Clinical and Experimental Medicine, Section of Hygiene and Occupational Health, University of Ferrara, Via Fossato Di Mortara, 24 44121 Ferrara, Italy; ^3^Department of Biomedical Sciences, Hygiene Section, University of Bari, Apulia Regional Epidemiological Observatory, Piazza Giulio Cesare 70100, Bari, Italy

## Abstract

Clinical and epidemiological surveillance of influenza and other Acute Respiratory Infections (ARI) are currently a major objective of Public Health. The aim was to describe the epidemiology of influenza using the Italian surveillance system. Vaccination Coverage (VC) rates were calculated during 1999-2009 influenza seasons. Molecular studies of influenza virus isolated, from patients with ILI, living in Apulia, are described. 1269 nasal-pharyngeal swabs were taken from patients with ILI and ARI in order to isolate and identify viruses using PCR. Influenza isolates are typed as being types A and B and influenza A isolates are A/H1 and A/H3. The progression of the ILI cases registered in Apulia was similar to the data recorded on a national level. The VC data recorded in Apulia showed a progressive increase in the vaccine doses administered to subjects over 65 years old. The virological surveillance showed a major circulation of the type A/H3N2 influenza virus during the peak incidence of the illness in seasons 1999-2000, 2002-2003, 2004-2005 and 2008-2009. During the same period, the lowest incidence was registered when the type A/H1N1 and B viruses were in circulation. In contrast, during the other seasons the lowest incidence was reported with A/H3N2 and B viruses.

## 1. Introduction

Influenza remains thus far a serious problem for many countries in connection with the worldwide spread of this disease [1]. Influenza virus infection is an important cause of respiratory infection in the population over the winter season, with peaks in Italy, from the end of December to the middle of March. Influenza strikes all age groups of the population but has a higher incidence in children and adolescents and causes a considerable increase in medical examinations and admissions to hospital. During epidemics, the highest number of complications and more than 90% of deaths associated with influenza are recorded in the elderly (>65 years old) [2, 3]. 

The virological basis of the frequent epidemics is related to the fact that influenza viruse can quickly mutate within their own antigenic structure. Changes can occur in surface glycolproteins, haemagglutinin and/or neuraminidase, producing new virus strains against with the population has no immunity [4]. For example, the recent emergence of H1N1 (swine flu) illustrates the ability of the influenza virus to create antigens new to the human immune system, even within a given hemagglutinin and neuraminidase subtype [5–7]. The high variability of influenza viruses makes it necessary to carry out comparative research of the antigenic and biological properties of epidemic influenza viruses [8]. Surveillance and monitoring the antigenicity of influenza viruses in circulation each year is necessary to identify any new variant strain so that vaccines can be updated annually [9]. 

In fact, there is an annual update of the composition of influenza vaccine and recommendations for vaccination against influenza according to clinical and epidemiological indications [10]. Although antiviral drugs are available for therapy and prevention, vaccination is still today the most effective preventive measure to control influenza and its complications [11, 12].

For these reasons the World Health Organization (WHO) has worked out a worldwide surveillance system on influenza to assess the antigenic changes of strains of the influenza virus circulating annually.

In Italy, epidemiological and virological surveillance of influenza is carried out by the Italian Net of Surveillance of Influenza (InfluNet) organized by the Italian National Institute of Health (ISS) and the Interuniversity Centre for Research on Influenza (CIRI) with the collaboration of the Regional Health Authorities. 

The system usually operates from October to April each year (from week 42 of each year and continued until week 17 of the following year) because influenza virus infection is an important cause of respiratory infection in the population over the winter season in Italy. For this reason, there hasn't been any attempt to isolate influenza virus in patients with respiratory symptoms during warmer months in Apulia.

The system is based on a network of volunteer sentinel GPs covering roughly 1% of the Italian population. Each sentinel practice records the daily number of consultations for Influenza Like Illness (ILI) and Acute Respiratory Infections (ARI), along with the patient's age group, on a standardised reporting form. The case definition used for ILI is an acute respiratory tract infection characterised by an abrupt onset of at least two of the following: fever, chills, headache, and myalgia. These data are collected by the local co-ordinator by e-mail, phone, or fax each Wednesday. 

Regional data join in defining epidemiological and virological national pictures that are weekly published on Italian Health Ministry website and reported by EISS network (European Surveillance Influenza Scheme).

Thus, just like in other European countries and the whole continent, it is possible to have, almost in real time, results on the Italian incidence of Influenza Like Illness (ILI) and Acute Respiratory Infections (ARI) by visiting the website: http://www.influciri.it/ [13–16]. 

Since 1999, the Laboratory of Hygiene, Department of Biological and Environmental Sciences and Technologies (Di.S.Te.B.A.) of the University of Salento, as part of the national programme of epidemiological and virological surveillance of influenza, is the Virological Reference Centre for Apulia.

In this paper, the results of the epidemiological and virological surveillance of influenza performed during 1999–2009 seasons in Apulia were presented and the relationship between age-specific morbidity rates and circulating strains were discussed. Also, the comparison between Apulia and Italian results was presented.

## 2. Subjects and Methods

### 2.1. Vaccination Coverage

In Italy, the target for vaccination coverage set by the national Health Plan (PNS) 1998–2000 and reiterated in the National Health Plan (NHP) 2005 is 75% in the population aged ≥65 years. Influenza Vaccination is provided free to subjects aged ≥65 years and to people at high risk (children aged 0–4 years, adults with chronic cardiovascular or respiratory diseases, patients with diabetes mellitus, etc.) according to the parameters provided by the annual circular issued by the Italian Ministry of Health. The vaccine is administered both through every AUSL (Azienda Unità Sanitaria Locale) of Apulia region and directly by general practitioners (GPs).

Actually, data relative to vaccination coverage in children and adults with chronic conditions are not available. For this reason, in the present paper, the rates of vaccination coverage for each season were estimated as follows:

(1) $$\begin{array}{cc} & \text{Vaccination}\;\text{coverage}\;\left(\text{\%}\right)\\ & \;\;=\frac{\text{Vaccinated}\;\text{individuals}\;\text{aged}\;\geq \;65\;\text{years}}{\text{Total}\;\text{population}\;\text{aged}\;\geq \;65\;\text{years}}\times 100. \end{array}$$

### 2.2. Epidemiological Surveillance

The Italian sentinel-practitioner-based Network for Surveillance of Influenza covers about 1% of the national population (56.996.000 ab).

In Apulia, during the ten influenza seasons studied, the surveillance started on week 42 of each year and continued until week 17 of the following year. During the surveillance period, influenza activity data (ILI or ARI cases), weekly collected by the sentinel doctors, were reported to the CIRI via electronic data transmission, detailing if subjects with influenza syndrome received vaccination. 

Rates for total incidence and those by age group (0–14, 15–64, and ≥65 years) are calculated using as a denominator the total number of patients assisted by physician participating in the study, because everyone has only a referent physician. Also, the seniors have only a referent physician and they aren't preferentially referred to the hospital. The propensity of each age group to visit the referent physician is related to the occurring of influenza syndrome.

**Figure 1:**
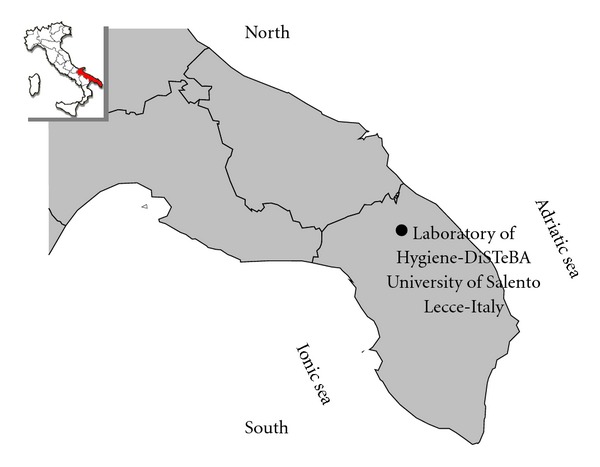
Map of Apulia.

In Apulia (4.068.167 ab), a total of 900 caring physicians and pediatricians report on a weekly basis the number of new cases of ILI according to a standard case definition: abrupt onset of fever (>38°C), one or more respiratory symptoms (nonproductive cough, sore throat, rhinitis) and one or more systemic symptoms (myalgia, headache, and severe malaise) (Figure 1). The number of ILI cases is used to calculate a rate per 1000 inhabitants and the total number of cases for the region, based on the proportion of the population serviced by the sentinel practitioners. 

The incidence of ILI was calculated every week as total incidence rate and age-group (0–15, 15–64, and ≥64 years) specific rate per 1000 surveilled subjects and the basis of the number of effectively reporting sentinels. The surveillance centre provides a weekly-updated web publication. The weekly incidence cut-off of 2‰  is considered to define the epidemic threshold. 

The incidence cut-off of 2 per 1000 per week was calculated to be the background level +3 standard deviations in all age groups and during the seasons examined. Background levels in each group were calculated by averaging weekly incidence rates in a 6-week period before and after the first and the last virus isolation, respectively. In order to calculate the age-specific proportion of subjects consulting for ILI during each season, weekly rates in age group during the epidemic period were accumulated, and accumulated background rates were subtracted, as previously described.

### 2.3. Virological Surveillance

In detail, during influenza seasons 1269 nasal-pharyngeal swabs were taken from patients with suspected influenza infections, randomly selected from collected samples during epidemiological surveillance, in order to isolate and identify viruses using RT-PCR.

The criteria for a laboratory identification of influenza are the isolation of the virus or the direct detection of viral antigen. Influenza isolates are typed as being types A and B, and influenza A isolates are further subtyped as being AH1 and AH3.

From 1999 to 2009, a RT-PCR was performed to identify influenza A and B viruses, RNA was extracted from samples using an adapted protocol for the RNeasy Mini Kit (Qiagen AG, Basel, Switzerland). cDNA was synthesised by reverse transcription; Nested PCR was performed using primers which amplified regions within the genes for (i) the influenza A matrix protein; (ii) the influenza B haemagglutinin (HA) (Influcheck Kit A/H3, Euroclone-Arrow Diagnostics S.r.l., Genova, Italy). From 2005 to 2009 this was replaced by a TaqMan Real-time PCR system in which both the reverse transcription and PCR reactions were performed in a single tube using primers and probes targeted at the highly conserved sequences in the A matrix gene and the influenza B HA gene (Fast set InfA/InfB—Arrow Diagnostics S.r.l., Genova, Italy). This is an improvement on our capacity to provide real-time surveillance to inform public health and policymakers.

## 3. Results

### 3.1. Vaccination Coverage

The rates of vaccination coverage for individuals aged ≥65 years old in Apulia during the ten influenza seasons studied are shown in Table 1. Influenza vaccination campaigns conducted among people over 65 years old during the last few years in Apulia have brought about a considerable increase in the vaccination coverage (VC) rates throughout the years studied. The national target of 75%, for subjects aged ≥65 years old, was reached only during 2005-2006 season (Table 1).

### 3.2. Epidemiological Surveillance

The clinical and epidemiological surveillance showed that the incidence and timing of influenza activity varied from season to season.

The weekly incidence cut-off of 2 per 1000 is considered to define the epidemic threshold.

Based on this threshold, the distribution of epidemic duration in Apulia started from week 46 of each year and continued until week 15 of the following year.

**Figure 2:**
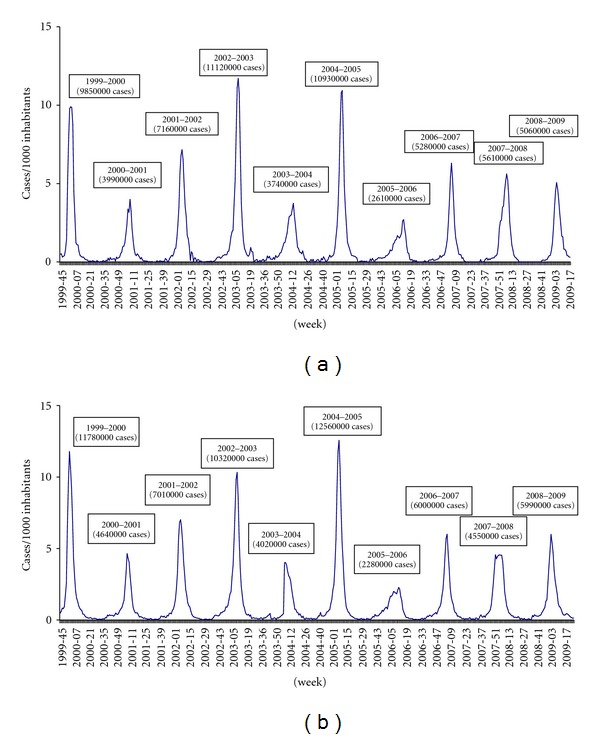
Epidemic curves related to the last ten seasons in Apulia (a) and in Italy (b).

In Apulia, the highest incidence rates occurred in the 2002-2003 and 2004-2005 seasons, with peaks, respectively, of 11 and 10.9 cases per 1000 subjects, which were significantly higher than those found in the other periods studied. A similar trend of incidence was observed in the last three seasons (Figure 2). 

In Table 2 we are summarizing the ILI rates of each season with the dominant subtype.

**Figure 3:**
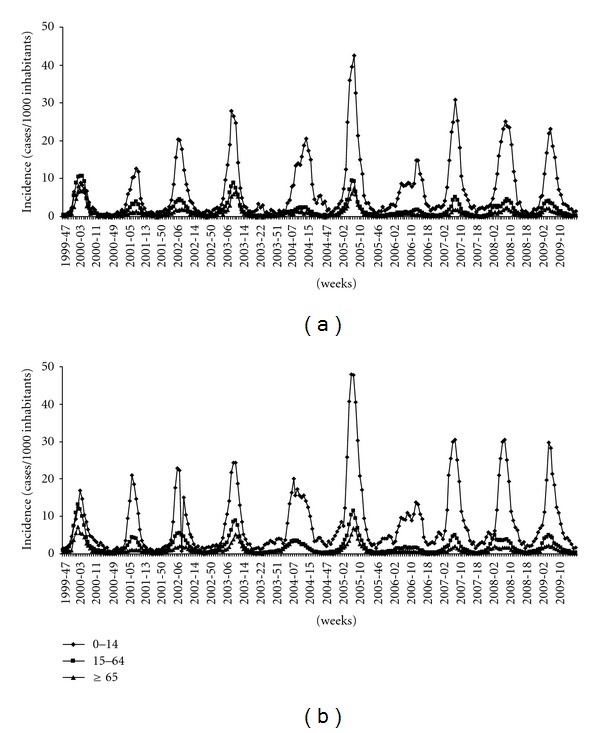
ILI morbidity in 0–14, 15–64, and ≥65 year-old subjects. A total case number for each season is estimated of the total cases for Apulia (a) and Italy (b).

During the study period (1999–2009), the ILI morbidity in 0–14 year-old subjects was higher than in adults and the elderly subjects (Figure 3). The active surveillance system showed the high annual burden of ILI among children and the low number of influenza vaccinated subjects in this age class. New preventive measures and strategies should be adopted to reduce the influenza related to morbidity and hospitalisations in paediatric population.

The progression of the ILI cases registered in Apulia during the period studied is similar to the data recorded on a national level (http://www.ministerosalute.it/). 

During the ten influenza seasons investigated, the progression of ARI showed, every year, a constant incidence throughout the period studied and a higher diffusion in the 0–14-year-age group. In fact, ARIs represent the most common childhood infection and are the main cause of medical consultations for children.

### 3.3. Virological Surveillance

**Figure 4:**
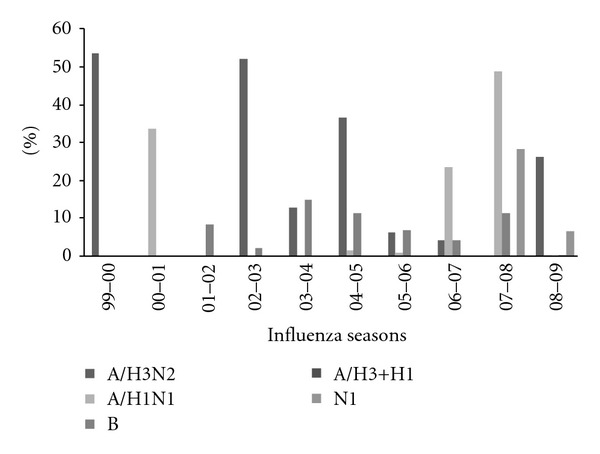
Percentage of Influenza virus type related to the last ten seasons in Apulia.

During the virological surveillance, nasopharyngeal samples were randomly collected from a relatively limited number of patients with suspected influenza syndrome. The typing of viruses isolated in the epidemic period from these samples showed the circulation of different virus strains (Figure 4).

In particular, in the 1999-2000 influenza season, all the viruses identified belonged to the A/H3N2 subtype (100%, 18/18). During the 2000-2001 season a A/H1N1 subtype virus was identified in all the samples tested (100%, 10/10). In the following season (2001-2002), all viruses belonged to type B (100%, 6/6). In the 2002-2003 season, there was a predominance of A/H3N2 influenza viruses (97%, 33/34), with a single type B. During 2003-2004 season 6/13 of the isolated viruses were A/H3N2, 7/13 (60%) were type B, while in 2004-2005 it was 30/31 (97%) A/H3N2 and only 1/31 (3%) type B. The 2005-2006 season was associated with circulation of influenza A/H3N2 and B (44% 8/18 and 50% 9/18, resp.), with minor participation of influenza A/H1N1 (6%). The epidemic influenza season in 2006-2007 was dominated by A/H1N1 (75%) with lower levels of A/H3N2 (12.5%) and type B (12.5%). During 2007-2008 season 16/32 (50%) of the isolated viruses were A/H1N1, 7/32 (22%) were type B, 9/32 (28%) were not yet identified subtype level. Finally, the epidemic influenza season 2008-2009 was dominated by A/H3N2 (83/108, 78%), with lower levels of B (3/108, 3%) and subtype A/H1N1 (1/108, 2%). In this season one sample was A/H3 + H1 subtype; 20/108 were not identified subtype level (17%).

## 4. Discussion

Molecular and antigenic data, together with the epidemiological findings, helps to determine age-specific morbidity rates during influenza epidemics. Collection of surveillance samples for influenza detection also assists in the monitoring of antigenic changes so that vaccines can be updated annually. Molecular epidemiological studies of influenza viruses in recent years have helped monitor genetic and antigenic drift and have also provided information on the evolutionary relationships and lineages of circulating strains [14, 16].

Anyway, it is well known that this surveillance tends to underestimate the actual number of cases. In fact, the incidence of influenza in the community is probably from three to six times higher, since not all cases go and see a general practitioner or paediatrician.

The VC data recorded in Apulia showed a progressive increase in the vaccine doses administered to subjects over 65 years old, even though the NHP levels have not been reached. The national target of 75%, for subjects aged ≥65 years old, was reached only during 2005-2006 season, probably due to the pandemic risk of avian flu (A/H5N1).

Vaccination Coverage rates also explain the lower incidence of ILI cases in the elderly.

The link between ILI rate's decline in seniors and the increase in vaccination coverage, from 40 to 75% in Apulia over the last decade, is obvious because in Italy influenza vaccination is provided free to subjects over 65 years.

In fact, the epidemic curve was different in the different age groups: the incidence rate was higher in children than in adults or in the age group ≥65 years old.

The progression of the ILI cases registered in Apulia during the period studied was similar to the data recorded on a national level.

During the 1999-2000 season, A/H3N2 viruses spread in all age groups, whereas in the following seasons a higher incidence was observed in the 0–14-year-age group, as expected, this group consists of subjects who neither were not exposed to previous influenza epidemics nor were vaccinated. Nevertheless, the data relative to the 1999-2000 season are attributable to the fact that during the first clinical and epidemiological surveillance season, Ps had not been involved as sentinel physicians, whereas their number progressively increased in the following years, improving the performance of the surveillance system. The highest rate of incidence in 0–14 years age group was registered during 2004-2005 season. 

The virological surveillance showed a major circulation of the type A/H3N2 influenza virus during the peak incidence of the illness in seasons 1999-2000, 2002-2003, 2004-2005, and 2008-2009. During the same period, the lowest incidence was registered when the type A/H1N1 and B viruses were in circulation. In contrast, during the seasons 2000-2001, 2001-2002, 2003-2004, 2005-2006, 2006-2007, and 2007-2008, the lowest incidence was reported when the type A/H3N2 and B viruses were in circulation.

Finally, during the 2008-2009 season, a cocirculation of A/H3 + H1 influenza virus was observed in a patient.

The progression of the ILI cases registered in Apulia during the period studied is similar to the data recorded on a national level with a slight delay; in fact the beginning of the epidemic period and the peak of the epidemic were registered always with a week of delay (6-7 days) in respect to the North of Italy. Instead, during the last influenza season, the beginning of epidemic period in Apulia was registered a month earlier (4 weeks) in respect to the national data, in fact in this region just before Christmas there was a spread of influenza.

The recent emergence of H1N1 (swine flu) illustrates the ability of the influenza virus to create antigens new to the human immune system, even within a given hemagglutinin and neuraminidase subtype. Triple-reassortant swine influenza A (H1) viruses, containing genes from avian, human, and swine influenza viruses, emerged and became an outbreak among humans worldwide. It has not reached that level yet, but physicians warn it could be worse in the fall when the expected return of swine flu mixes with the annual flu outbreak [5–7, 17]. 

In conclusion, the recent emergence of swine flu and the data obtained in these first ten years of activity highlight the importance of the network of clinical, epidemiological, and virological influenza surveillance as it helps the timely detection of epidemics and monitors their progress, provides early virus isolates to characterize the strains in circulation, provides a tool to estimate the effectiveness of vaccination campaigns, as well as being able to be combined with data from other regions to provide integrated national data.

## Figures and Tables

**Table 1:** Vaccination Coverage (VC) related to the last ten seasons in the elderly.

Influenza seasons	VC (%)
1999-2000	41.6
2000-2001	55.7
2001-2002	64.1
2002-2003	68.6
2003-2004	70.7
2004-2005	72.68
2005-2006	77.02
2006-2007	70.01
2007-2008	68.7
2008-2009	73.8

**Table 2:** ILI rates each season and dominant subtype.

Flu Season	Cumulative ILI rates	Dominant Subtype
1999-2000	70,64	H3N2
2000-2001	31,88	H1N1
2001-2002	56,53	B
2002-2003	83,72	H3N2
2003-2004	43,15	B
2004-2005	73,65	H3N2
2005-2006	31,86	B
2006-2007	41,98	H1N1
2007-2008	50,48	H1N1
2008-2009	40,20	H3N2
